# Serious Psychological Distress and Emergency Room Use among Adults with Multimorbidity in the United States

**DOI:** 10.1155/2017/8565186

**Published:** 2017-09-11

**Authors:** Khalid Alhussain, Abdulkarim M. Meraya, Usha Sambamoorthi

**Affiliations:** ^1^Department of Pharmaceutical Systems and Policy, School of Pharmacy, West Virginia University, P.O. Box 9510, Morgantown, WV 26505, USA; ^2^Clinical Pharmacy Department, Faculty of Pharmacy, Jazan University, Jizan 45142, Saudi Arabia

## Abstract

**Objectives:**

(1) To examine the association between serious psychological distress (SPD) and emergency room (ER) use in the past 12 months among adults with multimorbidity in the United States (US) and (2) to investigate the association between SPD and the reasons for ER use.

**Methods:**

The current study used a cross-sectional design with retrospective data from the 2015 National Health Interview Survey. Logistic regression models were used to assess the association between SPD and ER use among adults with multimorbidity. Among ER users, adjusted logistic regression models were conducted to examine the association between SPD and the reasons for the ER use.

**Results:**

After controlling for other variables, adults with multimorbidity and SPD were more likely to use ER than those with multimorbidity and no SPD (AOR = 1.61, 95% CI = 1.26, 2.04). Among ER users, there were no significant associations between SPD and the reasons for ER use after controlling for other variables.

**Conclusion:**

Adults with multimorbidity and SPD were more likely to use ER as compared to those with multimorbidity and no SPD. Among adults with multimorbidity, routine screening for SPD may be needed to reduce the ER use.

## 1. Introduction 

The prevalence of multimorbidity, the coexisting of two or more chronic illnesses in the same individual has grown in the United States (US) [1]. In 2012, one in four civilian, noninstitutionalized US adults had multimorbidity [2]. Multimorbidity is associated with high healthcare utilization and cost [3, 4]. It is also associated with poor physical function [5], poor health-related quality of life (HRQoL) [6], and poor mental health, specifically, serious psychological distress (SPD) [7, 8]. Adults with multimorbidity are more likely to have SPD as previous studies showed [7, 8].

Multimorbidity as well as SPD has been shown to be associated with high healthcare utilization, especially, emergency room (ER) use [9–12]. Previous studies have indicated that multimorbidity was positively associated with ER use [10, 11]. In a study assessing the association between multimorbidity and ER use, adults who had higher Charlson Comorbidity Index (CCI) scores were more likely to use the ER [11]. Further, there is a positive association between SPD and ER use [9, 12]. One study using the 2004–2006 National Interview Health Survey data indicated that adults with SPD were 2.05 times as likely to use the ER as adults without SPD [12]. Another study using the 2007 Medical Expenditure Panel Survey data found that adults with SPD had higher ER visits than those without SPD [9].

However, no study has examined the use of ER among adults who suffer from both multimorbidity and SPD. Moreover, the reasons behind the possible association between SPD and ER use among adults with multimorbidity have not been investigated yet. Based on prior literature [9–12] we can speculate that adults with multimorbidity and SPD may be more likely to use ER because of the complexity of healthcare management as well as access issues. For example, adults with multimorbidity and SPD may use ER because of seriousness of their conditions. Therefore, the first objective of this study was to examine the association between SPD and ER use among US adults with multimorbidity and the second objective was to examine the association between SPD and the reasons for ER use.

## 2. Methods

### 2.1. Conceptual Framework

We used the expanded Andersen healthcare utilization model [13]. The purpose of this model is to demonstrate the factors that may lead to the use of ER. Based on this model, ER use may be influenced by five domains: predisposing factors (e.g., age and sex), enabling factors (e.g., education level and health insurance), need factors (e.g., SPD status and functional limitations), personal health practices (e.g., smoking status and alcohol use), and external environmental factors (e.g., the region of residence).

### 2.2. Study Design

We adopted a retrospective cross-sectional study using National Health Interview Survey (NHIS).

### 2.3. Data Source

The current study used data from 2015 NHIS, US nationally representative data. NHIS is an annual, cross-sectional survey among the civilian noninstitutionalized population of the US. To select the sample of dwelling units for the NHIS, multistage sampling techniques were used. Multistage methods divided the target universe into several nested levels of strata and clusters. This survey consists of two main sections, core and supplements. This study used data from the family, person, sample adult core, and imputed income files. Sociodemographic characteristics, health status, and health insurance were captured from the person file. Family file provided information about the poverty status of the household. The sample adult core file provided information about chronic physical conditions, psychological distress, access to care, and utilization of healthcare services [14].

### 2.4. Study Sample

The study sample was based on US adults aged >21 years having multimorbidity (*N* = 12,910). Multimorbidity was derived from the sample adult core file. It was defined as the presence of two or more of the following chronic physical conditions: arthritis, asthma, cancer, hepatitis, kidney disease, chronic obstructive pulmonary disease (COPD), diabetes, heart diseases (angina pectoris, coronary heart disease, heart attack, and other heart conditions), hyperlipidemia, hypertension, or stroke. The list of chronic conditions was adapted from a previous study [15]. We also excluded a few individuals with missing data in health insurance (*N* = 25) and ER use (*N* = 16). Thus, the final sample size consisted of 12,869 adults with multimorbidity.

### 2.5. Measures

#### 2.5.1. Dependent Variables

*(1) Emergency Room Use*. ER use was self-reported and derived from the sample adult core file. Adults were asked, “During the past 12 months, how many times have you gone to a hospital ER about your own health?” Those who reported at least one ER visit were considered as “ER users.” 

*(2) Reasons for the Most Recent ER Visit*. Reasons for the most recent ER visit were extracted only for adults who had reported using ER in the past 12 months. In the NHIS, ER users were asked several questions about the reasons for their most recent ER visit. For this study, we categorized the reasons for the most recent ER visit into three categories based on a previous study [16]. First, the “lack of access to other providers” category included adults who selected at least one of the following reasons “did not have another place to go,” “emergency room is the closest provider,” or “get most of their care at the emergency room.” Second, the “seriousness of the medical problem” category contained adults who reported one of the following reasons to visit ER: “health provider advised to go,” “the problem was too serious for the doctor's office or clinic,” “only a hospital could help,” or “arrived by ambulance or another emergency vehicle.” Third, the “doctor's office or clinic was not open” category included those who reported “doctor's office or clinic was not open.” It has to be noted that respondents can provide multiple reasons for their recent ER visit.

#### 2.5.2. Key Independent Variable: SPD Status (SPD or No SPD)

SPD was measured by the Kessler-6 (K6) [17]. The K6 asks participants to assess the six symptoms of psychological distress over the past 30 days with a response scale ranging from 0 which is none of the time to 4 which is all of the time. The symptoms include feeling nervous, hopeless, restless or fidgety, so depressed that nothing could cheer you up, and worthless. The total K6 score ranges from 0 to 24, with higher scores indicating more severe psychological distress. A dichotomous variable indicating SPD or no SPD was constructed. Based on previous research, SPD was defined as a score of 13 or greater on the K6 since this cut-off is an indicator for mental illnesses [18].

#### 2.5.3. Other Independent Variables

Predisposing factors included age (22–39 years, 40–49 years, 50–64 years, and 65 years and older), sex, and race/ethnicity (Hispanic, non-Hispanic white, non-Hispanic black, and other non-Hispanic races). Enabling factors included marital status (married, widowed/divorced/separated, and never), education (less than high school, high school, and greater than high school), poverty status (poor (less than 100% federal poverty line), near poor (100% to less than 200%), middle (200% to less than 400%), and high income (greater than or equal to 400%)), and health insurance. Health insurance was defined as insured and uninsured. Need factors included SPD status (SPD, no SPD), health status (excellent/very good, good, and fair/poor), and functional limitations (yes/no). Personal health practices comprised smoking status (nonsmoker, former smoker, and current smoker), alcohol use (lifetime abstainer, former drinker, and current drinker), physical activity (daily, weekly, no exercise, and unable), and body mass index (BMI). BMI categories were classified into three classifications (underweight/normal (0–25.0 kg/m^2^), overweight (25.0–30.0 kg/m^2^), and obese (30.0–40.0 kg/m^2^)). Finally, external environmental factor was the region of residence (Northeast, Midwest, South, and West region).

### 2.6. Statistical Analyses

Chi-square tests were used to examine the associations between SPD status and the independent variables in the bivariate analysis. Adjusted logistic regression models were conducted to examine the association between SPD status and ER use. In these regressions, predisposing factors, enabling factors, need factors, personal health practices, and external environmental factors were included as independent variables. Among ER users, separate adjusted logistic regression models were conducted to examine the association between SPD status and the reasons for the ER use in the past 12 months. In these regressions, predisposing factors, enabling factors, need factors, personal health practices, and external environmental factors were included as independent variables.

## 3. Results

### 3.1. Description of the Study Participants

The characteristics of the study participants (*N* = 12,869) are presented in Table 1. Among adults with multimorbidity, 5.8% had SPD and 94.2% did not. More than half of adults with multimorbidity and SPD used the ER in the past 12 months. Nearly a quarter of adults with multimorbidity and no SPD used the ER in the past 12 months. The majority of adults with multimorbidity and SPD (92%) had functional limitations, whereas 61.3% of those with multimorbidity and no SPD had functional limitations. About 70% of adults with multimorbidity and SPD reported fair/poor general health and only 23.7% of those with multimorbidity and no SPD reported fair/poor general health. We also found that 31.7% of adults with multimorbidity and SPD had poor income. In contrast, about 10% of adults with multimorbidity and no SPD had poor income. Statistically significant differences were also observed in sex, age, race/ethnicity, marital status, education level, health insurance, smoking status, alcohol use, and physical activity between adults with multimorbidity and SPD and those with multimorbidity and no SPD (see Table 1).

### 3.2. SPD and ER Use

Table 2 displays weighted row percentages, adjusted odds ratios (AOR), and 95% confidence intervals (CI) from multivariable logistic regression. A statistically significant association (<0.001) between SPD and ER use in the past 12 months was observed. A higher proportion of adults with multimorbidity and SPD reported using ER in the past 12 months compared to those with multimorbidity and no SPD (54.1% versus 25.2%). In multivariable logistic regression, after controlling for predisposing factors, enabling factors, need factors, personal health practices, and external environmental factors, the association between SPD and ER use remained statistically significant. Adults with multimorbidity and SPD were 1.61 (95% CI = 1.26, 2.04) times as likely to use ER in the past 12 months as those with multimorbidity and no SPD.

### 3.3. Reasons for the Most Recent ER Visit among ER Users

The majority of ER users (84.2%) reported seriousness of medical problems as the reason for the most recent ER visit. About 47% reported doctor's office or clinic was not open and 54.8% reported lack of access to other providers as the reason for the most recent ER visit. In the bivariate analysis, we found a statistically significant association between SPD and “lack of access to other providers” as the reason for the most recent ER visit. In multivariable logistic regression, after controlling for predisposing factors, enabling factors, need factors, personal health practices, and external environmental factors, there were no statistically significant associations between SPD and the reasons for the most recent ER visit including “lack of access to other providers” (see Table 3). However, a closer examination of the results revealed that without controlling for the age and health insurance in the logistic regression model, adults with multimorbidity, and SPD were 1.42 times (95% CI = 1.03, 1.96) as likely to report “lack of access to other providers” as the reason for the most recent ER visit compared to those with multimorbidity and no SPD. Once the age and health insurance were entered in the model, the statistical significance of SPD disappeared.

## 4. Discussion

We examined the relationship between SPD and ER use among adults with multimorbidity. Our results revealed that there was a statistically significant relationship between SPD and ER use. Adults with multimorbidity and SPD were more likely to use ER compared to adults with multimorbidity and no SPD. This is consistent with the previous studies showing that SPD is positively associated with ER use among general population [9, 12]. Several reasons could explain the higher likelihood of ER use among adults with multimorbidity and SPD as compared to those with multimorbidity and no SPD. For example, our study findings indicate that adults with multimorbidity and SPD are at high risk for disability and poor health. This has been documented in published literature as well [19, 20]. Further, adults with SPD were more likely to avoid visiting a doctor due to the fear of having a serious disease, which may lead to complications that require ER care [21]. Again, lack of mental health-related treatment can lead to ER use. For example, in 2009, nearly 50% of US adults with SPD did not receive healthcare services for mental illness [9].

In our study, 54.2% of adults with multimorbidity and SPD used ER during the past 12 months, which is a cause for concern. Adults with multimorbidity and SPD need care from both chronic condition specialists and mental health specialists [22]. Seeking care in an ER setting may not be effective for these individuals because of limited number of trained ER healthcare professionals who can provide care for mental illness [23]. Given that treatment of chronic conditions in ER is very expensive in the US [24], there is a need to develop health policy programs targeting those with multimorbidity and SPD.

Our findings have implications for promoting collaborative care to reduce ER use. Caring for adults with multimorbidity and SPD is challenging because of competing demands and shifting priorities [25]; their care may be compromised due to lack of care coordination between the mental health and physical health providers [22]. Under fee-for-service healthcare systems, the lack of communication and coordination of care between mental health and physical health professionals has been well documented [22]. Such lack of coordination may lead to higher ER use among those with multimorbidity and SPD. To address these issues, collaborative care model may help in managing adults with multimorbidity and SPD. Numerous studies have provided evidence that collaborative care interventions can lead to better outcomes for those with physical and mental illnesses [26, 27]. One study evaluating the role of collaborative care for individuals with chronic diseases and depression found that individuals who received collaborative care had better chronic disease outcomes than those who received usual care [26].

Among adults with multimorbidity, the majority of ER users (84.2%) reported seriousness of medical problems as the reason for using ER, which has implications for all adults with multimorbidity. Complexity and seriousness of medical problems among those with multimorbidity are well documented [1]. To prevent ER use among those with multimorbidity in general, prevention strategies may need to be emphasized. For example, for many of the chronic conditions included in our list (e.g., heart disease, diabetes, COPD, and stroke), complications can be easily avoided through modification of life-style risk factors. According to the Centers for Disease Control and Prevention, four modifiable health risk behaviors—lack of exercise, poor nutrition, tobacco use, and excessive alcohol use—have accounted for much of the complications related to chronic disease [28]. These factors may also be relevant for those with multimorbidity. Also, these four modifiable risk factors have been shown to increase ER use [29–31]. Therefore, to reduce ER use among those with multimorbidity, secondary prevention strategies need to target these four modifiable risk factors [28].

Our study had both strengths and limitations. One of the strengths was that the study used nationally representative data for adults with multimorbidity. Furthermore, the association between SPD and ER use was examined after controlling for predisposing, enabling, need, personal health practices, and environmental factors. Another strength was that the reasons behind ER use were investigated. The study findings have to be interpreted in the context of its limitations. We adopted a cross-sectional study design, which can only suggest associations. All data were self-reported and may be subject to recall bias. The definition of multimorbidity was limited. We did not examine the intensity of ER use (i.e., number of ER visits). We only assessed the reasons for the most recent ER visit.

## 5. Conclusion

The present study found that adults with multimorbidity and SPD were more likely to use ER as compared to those with multimorbidity and no SPD. Given the high risk of SPD among adults with multimorbidity, routine screening for SPD may be needed. Also, collaborative care model may be needed to minimize the risk of ER use among those with multimorbidity and SPD.

## Figures and Tables

**Table 1:** Characteristics of adults with multimorbidity by serious psychological distress status. National Health Interview Survey, 2015.

All	Total sample		SPD		No SPD		*P* value
	*N*	Wt.%	*N*	Wt.% (Col%)	*N*	Wt.% (Col%)	
	12,869	100.0	772	5.8	12,097	94.2	
ER use							<0.001
Yes	3,663	26.8	412	54.1	3,251	25.2	
No	9,206	73.2	360	45.9	8,846	74.8	

*Predisposing factors*							
Sex							<0.001
Women	7,350	53.0	509	62.8	6,841	52.4	
Men	5,519	47.0	263	37.2	5,256	47.6	
Age in years							<0.001
22–39 years	1,131	10.2	108	19.0	1,023	9.7	
40–49 years	1,394	12.4	144	16.8	1,250	12.1	
50–64 years	4,241	36.3	333	44.0	3,908	35.8	
65 and older	6,103	41.1	187	20.2	5,916	42.3	
Race/ethnicity							0.012
Hispanic	1,480	10.4	139	15.3	1,341	10.1	
Non-Hispanic white	8,859	72.8	500	69.1	8,359	73.1	
Non-Hispanic black	1,889	11.9	95	10.5	1,794	12.0	
Other non-Hispanic races	641	4.9	38	5.1	603	4.9	

*Enabling factors*							
Marital status							<0.001
Married	6,165	63.0	279	47.5	5,886	64.0	
Widowed, separated, divorced	5,135	27.5	356	35.7	4,779	27.1	
Never	1,549	9.4	134	16.9	1,415	8.9	
Education level							<0.001
Less than high school	2,104	14.5	223	29.8	1,881	13.6	
High school	3,559	27.3	210	26.9	3,349	27.5	
Greater than high school	7,149	57.7	337	43.2	6,812	58.9	
Poverty status							<0.001
<100% FPL	1,945	11.1	295	31.5	1,650	9.9	
100–<200%	2,748	18.5	218	27.2	2,530	17.9	
200–<400%	3,474	26.5	153	24.4	3,321	26.6	
≥400%	3,848	37.0	74	12.1	3,774	38.6	
Missing	854	6.9	32	4.8	822	7.0	
Health insurance							<0.001
Insured	12,228	95.0	691	86.6	11,537	95.5	
Uninsured	641	5.0	81	13.4	560	4.5	

*Need factors*							
Perceived general health							<0.001
Excellent/very good	4,737	39.0	76	11.9	4,661	40.6	
Good	4,486	34.7	158	19.2	4,328	35.7	
Fair/Poor	3,642	26.3	538	68.9	3,104	23.7	
Functional limitations							<0.001
Yes	8,547	63.0	715	92.0	7,832	61.3	
No	4,312	36.9	57	8.0	4,255	38.7	

*Personal health practices*							
Body mass index							0.008
Underweight /normal	3,223	24.0	197	26.1	3,026	23.9	
Overweight	4,246	33.3	193	26.3	4,053	33.8	
Obese	5,076	39.8	354	42.9	4,722	39.6	
Missing	324	2.8	28	4.7	296	2.7	
Smoking status							<0.001
Nonsmoker	6,521	51.7	285	37.4	6,236	52.6	
Former smoker	4,210	32.8	166	20.5	4,044	33.6	
Current Smoker	2,126	15.4	321	42.1	1,805	13.8	
Alcohol use							<0.001
Lifetime abstainer	2,581	17.9	143	18.2	2,438	18.0	
Former drinker	3,088	22.9	249	32.1	2,839	22.4	
Current drinker	7,125	58.7	376	49.7	6,749	59.6	
Physical activity							<0.001
Daily	623	4.9	25	2.7	598	5.1	
Weekly	2,825	24.8	75	11.2	2,750	25.8	
No exercise	8,811	66.2	586	75.3	8,225	65.9	
Unable	565	3.7	82	10.7	483	3.3	

*External environmental factors*							
Region of residence							0.615
Northeast	2,197	17.6	132	15.1	2,065	17.8	
Midwest	2,768	23.2	155	23.9	2,613	23.2	
South	4,603	38.6	287	40.6	4,316	38.5	
West	3,301	20.6	198	20.4	3,103	20.6	

*Note*. Based on 12,869 adults aged over 21 years having two or more physical diseases with and without serious psychological distress (SPD). SPD was defined as a score of 13 or greater on the K6. SPD: serious psychological distress; ER: emergency room; Wt.: weighted; FPL: federal poverty line.

**Table 2:** Weighted row percentages, adjusted odds ratios, and 95% confidence intervals of serious psychological distress from logistic regressions on ER use in the past 12 months. National Health Interview Survey, 2015.

	Wt.%	AOR	95% CI	*P* value
*Predisposing factors*				
Sex				
Women	27.9	0.98	[0.86, 1.10]	0.685
Men (Ref.)	25.6			
Age in years				
22–39	39.3	2.70	[2.21, 3.30]	<0.001
40–49	27.0	1.35	[1.13, 1.62]	0.001
50–64	25.9	1.14	[1.00, 1.30]	0.058
≥65 (Ref.)	24.5			
Race/ethnicity				
Hispanic	29.6	0.93	[0.77, 1.13]	0.487
Non-Hispanic black	35.1	1.34	[1.15, 1.56]	<0.001
Other non-Hispanic races	19.0	0.66	[0.51, 0.84]	0.001
Non-Hispanic white (Ref.)	25.6			

*Enabling factors*				
Marital status				
Married	24.0	1.08	[0.89, 1.30]	0.448
Widowed, separated, divorced	31.0	1.18	[0.97, 1.42]	0.090
Never (Ref.)	33.7			
Education level				
High school	28.8	0.92	[0.76, 1.13]	0.436
Greater than high school	23.4	0.93	[0.77, 1.13]	0.464
Less than high school (Ref.)	36.8			
Poverty status				
100–<200%	34.9	0.89	[0.74, 1.06]	0.203
200–<400%	26.7	0.72	[0.59, 0.87]	0.001
≥400%	18.3	0.65	[0.53, 0.80]	0.000
Missing	26.1	0.83	[0.63, 1.08]	0.169
<100% FPL (Ref.)	42.9			
Health insurance				
Insured	26.4	1.00	[0.78, 1.28]	0.999
Uninsured (Ref.)	35.7			

*Need factors*				
SPD status				
SPD	54.1	1.61	[1.26, 2.04]	<0.001
No SPD (Ref.)	25.2			
Perceived general health				
Excellent/very good	15.8	0.40	[0.34, 0.48]	<0.001
Good	26.1	0.62	[0.54, 0.71]	<0.001
Fair/poor (Ref.)	44.1			
Functional limitations				
No	16.0	0.55	[0.48, 0.64]	<0.001
Yes (Ref.)	33.2			

*Personal health practices*				
Body mass index				
Underweight/normal	28.6	1.18	[1.02, 1.38]	0.028
Overweight	22.9	0.92	[0.81, 1.05]	0.231
Missing	23.7	0.76	[0.54, 1.09]	0.133
Obese (Ref.)	29.3			
Smoking status				
Nonsmoker	23.5	0.77	[0.66, 0.91]	0.001
Former smoker	26.4	0.88	[0.75, 1.04]	0.145
Current smoker (Ref.)	38.7			
Alcohol use				
Lifetime abstainer	29.3	1.10	[0.92, 1.31]	0.281
Former drinker	32.6	1.16	[1.00, 1.34]	0.047
Current drinker (Ref.)	23.9			
Physical activity				
Daily	19.3	0.75	[0.56, 1.01]	0.059
Weekly	19.2	0.87	[0.74, 1.02]	0.082
Unable	47.4	1.51	[1.13, 2.01]	0.005
No exercise (Ref.)	29.0			

*External environmental factors*				
Region of residence				
Northeast	26.2	1.03	[0.87, 1.23]	0.714
Midwest	28.3	1.08	[0.93, 1.26]	0.306
South	27.1	0.95	[0.83, 1.08]	0.414
West (Ref.)	25.1			

*Note*. Based on 12,869 adults aged over 21 years having two or more physical diseases with and without serious psychological distress (SPD). SPD was defined as a score of 13 or greater on the K6. Wt.: weighted; AOR: adjusted odds ratio; CI: confidence interval; Ref.: reference group; SPD: serious psychological distress; FPL: federal poverty line.

**Table 3:** Weight row percentages, adjusted odds ratios, and 95% confidence intervals of serious psychological distress from separate logistic regressions on reasons of the recent ER use in the past 12 months. Adults with multimorbidity and ER use. National Health Interview Survey, 2015.

SPD status	Wt.%	AOR	95% CI	*P* value
*Seriousness of medical problem*				
SPD	85.6	1.03	[0.65, 1.63]	0.888
No SPD (Ref.)	84.0			

*Doctor's office or clinic was not open*				
SPD	44.9	0.93	[0.67, 1.29]	0.676
No SPD (Ref.)	46.4			

*Lack of access to other providers*				
SPD	67.0	1.27	[0.91, 1.76]	0.165
No SPD (Ref.)	53.3			

*Note*. Based on 3,663 adults aged over 21 years having two or more physical diseases with and without serious psychological distress (SPD). SPD was defined as a score of 13 or greater on the K6. Adjusted model included sex, age, race/ethnicity, marital status, education level, poverty status, perceived general health, functional limitations, body mass index (kg/m^2^), smoking status, alcohol use, physical activity, and region of residence. Wt.: weighted; AOR: adjusted odds ratio; CI: confidence interval; Ref.: reference group; SPD: serious psychological distress.
